# Adapting Enhance®Fitness for People Living With Dementia and their Care Partners through a RE-AIM Lens

**DOI:** 10.1093/geroni/igaf122.4058

**Published:** 2025-12-31

**Authors:** Sarah McKiddy, Wenting Peng, Basia Belza

**Affiliations:** University of Washington, Seattle, Washington, United States; University of Washington, Seattle, Washington, United States; University of Washington, Seattle, Washington, United States

## Abstract

Multidomain lifestyle trials demonstrate that combined behavior change can support cognitive health. The FINGER demonstrated that a two-year multidomain lifestyle intervention helped maintain or improve cognition compared to general health advice in older adults who had elevated dementia risk factors. Preliminary findings from the U.S. POINTER trial similarly suggests that a multidomain approach led to greater cognitive benefits than self-guided health promotion among adults at elevated dementia risk. Despite these promising studies, widely disseminated community-based exercise programs have rarely been adapted for, or rigorously tested with, people living with dementia (PLWD) and their care partners. Enhance®Fitness (EF) – a long-standing, evidence-based, low-cost group exercise and falls prevention program with broad adoption across community and health system settings in the U.S. – improves physical performance and demonstrates strong participant retention. Remotely delivered EF has proven feasible for older adults with arthritis, and a pragmatic trial is comparing in-person and tele-EF. Yet many EF studies have excluded participants with cognitive impairment and no EF study examined the effectiveness of improving cognitive function, limiting relevance to PLWD. This presentation explores considerations for tailoring EF to support PLWD and their care partners. Embedding EF in dementia prevention trials would allow evaluation of outcomes meaningful to PLWD and families, including social engagement and caregiver support. The RE-AIM framework guides our evaluation to identify facilitators and barriers specific to PLWD and families. EF’s national infrastructure and interdisciplinary research base offers a robust pathway to extend dementia-friendly lifestyle interventions to community-based health promotion programs.

